# Memory Effects on Movement Behavior in Animal Foraging

**DOI:** 10.1371/journal.pone.0136057

**Published:** 2015-08-19

**Authors:** Chloe Bracis, Eliezer Gurarie, Bram Van Moorter, R. Andrew Goodwin

**Affiliations:** 1 Quantitative Ecology and Resource Management, University of Washington, Seattle, Washington, United States of America; 2 Senckenberg Biodiversity and Climate Research Centre (BiK-F), Senckenberg Gesellschaft für Naturforschung and Goethe Universität Frankfurt, Frankfurt (Main), Germany; 3 Department of Biology, University of Maryland, College Park, Maryland, United States of America; 4 Centre for Conservation Biology, Norwegian University of Science and Technology, Trondheim, Norway; 5 Norwegian Institute for Nature Research (NINA), Trondheim, Norway; 6 Environmental Laboratory, US Army Engineer Research and Development Center, Portland, Oregon, United States of America; Università degli Studi di Napoli Federico II, ITALY

## Abstract

An individual’s choices are shaped by its experience, a fundamental property of behavior important to understanding complex processes. Learning and memory are observed across many taxa and can drive behaviors, including foraging behavior. To explore the conditions under which memory provides an advantage, we present a continuous-space, continuous-time model of animal movement that incorporates learning and memory. Using simulation models, we evaluate the benefit memory provides across several types of landscapes with variable-quality resources and compare the memory model within a nested hierarchy of simpler models (behavioral switching and random walk). We find that memory almost always leads to improved foraging success, but that this effect is most marked in landscapes containing sparse, contiguous patches of high-value resources that regenerate relatively fast and are located in an otherwise devoid landscape. In these cases, there is a large payoff for finding a resource patch, due to size, value, or locational difficulty. While memory-informed search is difficult to differentiate from other factors using solely movement data, our results suggest that disproportionate spatial use of higher value areas, higher consumption rates, and consumption variability all point to memory influencing the movement direction of animals in certain ecosystems.

## Introduction

Sustaining animal populations in fragmented landscapes often depends on understanding and managing their movements, but a major challenge comes from individuals changing their response to a stimulus depending on the context of their decision [1, 2]. An event can therefore elicit variable animal responses as an individual’s context changes based on past experiences, even in seemingly static or unchanging environments. Learning and memory are key factors in the emergence of decision context. The unique experience of an individual can change its perceived value of an environmental stimulus over time, which can shape the decisions that underlie an observed movement trajectory [1, 3].

A range of taxa, from insects to primates, have been shown to exhibit spatial memory during foraging. Bees, for example, learn reward values [4]. When displaced into familiar territory, they can choose between two goals and navigate to that goal, demonstrating a map-like memory [5], although path integration and learned landmarks may be sufficient to accomplish these tasks [6]. Fish on coral reefs use learned landmarks to navigate between food patches, including the possibility of novel routes indicating a cognitive map [7]. Birds repeatedly return to previously visited sites [8]. An analysis of visit length and time between visits suggests that monkey movements are non-random due to the use of memory and visitation patterns are driven by resource availability [9].

Studies of foraging behavior in the field have provided evidence for learning and memory across a variety of taxa, complementing the study of foraging and memory in controlled laboratory studies (reviewed in [10]). Memory may be key to understanding patterns observed in animal foraging, yet memory-informed search is difficult to differentiate from other sensory-driven search behaviors [11, 12]. Unlike physiological attributes such as energy reserves or hormone levels, memory is an internal state of the animal that cannot be measured directly. This is especially true in ecological contexts when the history of the animal’s experiences may be unknown. In fact, formulating clear behavioral criteria to infer cognitive processes is a particular challenge [13]. Simulation models are therefore a key analytical tool to investigate hypotheses that involve direct measures or manipulation of memory.

Prior work suggests that in foraging, memory-informed movement is advantageous in predictable landscapes [14] and therefore negatively correlated with the rate of environmental change [15]. Incorporating memory has been suggested as a future direction for improving movement models [16, 17]. Methods based on random walks or area-restricted search are often used to interpret movement [11, 18, 19]. When memory is incorporated, the models are often system-specific [20, 21] or use an homogeneous landscape [22, 23], ignoring that heterogeneity in habitat quality can also affect revisit probability [9]. Other studies suggest memory is important to home range formation, which emerges from animals learning their environment as an intermediate state between dispersive and site-fidelious tendencies [24–26]. Returning to a location is a function of information decay and distance: either the balance between a repulsive working memory and an attractive reference memory decaying at different rates and weighted by distance [24], or when foragers benefit from site-specific information after a time delay, such that there is a dynamic equilibrium between resource consumption and regeneration [25].

We explored conditions under which the added cognitive complexity of maintaining memory is advantageous to foraging by creating a flexible, continuous model incorporating memory for an animal moving through a heterogeneous landscape. The main strategic objectives in constructing the model were (1) to build a nested sequence of increasingly more complex models from a random walk null model, (2) to have a straightforward currency for comparing outcomes that, in this case, is resource consumption, and (3) to make the model continuous in space and time to explore properties across scales without concern for the effect of discretization. To illustrate the model, we compare an animal using three nested movement processes: (1) a simple correlated random walk, (2) kinesis, in which correlated random walk parameters, i.e., searching and feeding behaviors, change as a function of the consumption rate, and (3) memory-informed movement, in which directional bias in the searching behavior is informed by previous experiences. We compare the three movement processes across a range of landscape characteristics (patchiness, spatial correlation, and regeneration rate).

To facilitate comparisons of the benefit of the memory process at various interactions of scales, particularly the spatial scales of the landscape with the spatial scale of foraging and memory, we use continuous processes with similar underlying Gaussian kernels for the movement model, the landscape representation, and the foraging and memory processes. This makes the prototypical scenario examined here an animal grazing across stationary resources that deplete and regenerate, rather than a predator consuming discrete prey.

These nested movement processes (random walk, kinesis, and memory) allowed us to evaluate the landscape characteristics that result in the memory model outperforming other behaviors, with implications for the evolution of memory [27]. We were also interested in how the spatial and temporal scales of the memory process relate to the scale of patterns in the landscape, particularly the resource regeneration rate that affects the preferred timing of patch revisits. To better understand the reasons underlying differences among movement processes, we analyzed the corresponding behavioral differences in terms of habitat use and time allocation among the movement processes, and what they suggest for patterns one is likely to observe for an animal using memory.

## Methods

In our model, a simulated individual moves through a habitat of variable resource quality, consuming those resources. The individual’s movements may be random (random walk), informed by its current consumption rate (kinesis), or informed by its memory of resource quality and consumption rate (memory). In the framework of Nathan et al. [28], finding food resources motivates the animal’s movement, and its internal goal is to maximize consumption. We vary only the animal’s directional tendency; all other model attributes are similar across movement processes. While the random walk model has no external influence, the current resource quality influences the kinesis movement process. In memory-based movement, both current resource quality and spatial memory influence movement, and navigation towards previously learned habitat areas is possible. The model is two-dimensional in space *z* ∈ *R* ^2^ with time *t* (e.g., *f*(*z*, *t*), though the dependent variables *z* and *t* are omitted in some equations for clarity). Parameters and symbols are listed in Table 1.

**Table 1:** Parameters used in the foraging model and values for simulations. Because units are arbitrary in the simulations, L is used for generic length units and T is used for generic time units.

Parameter	Definition	Units	Values
Simulations			
Δ*t*	model time step	T	0.1
*T*	simulation length (time steps)		1000
Landscapes			
*μ*_*Q*_	patch concentration (GRF mean)		-1.5, -1, -0.5, 0, 1
*γ*_*Q*_	patch size (GRF scale)		2, 10
Consumption			
*β*_*R*_	regeneration rate	1/T	0.005, 0.01, 0.05
*β*_*C*_	consumption rate	1/T	1
*γ*_*C*_	consumption spatial scale	L	1
Memory^a^			
*ψ*_*M*_	short-term memory factor		2, 5, 10
*β*_*L*_, *β*_*S*_	learning rates	1/T	1
*ϕ*_*L*_, *ϕ*_*S*_	decay rates	1/T	0, 1e-5, 1e-4, 0.001, 0.01, 0.1, 0.5
*γ*_*L*_, *γ*_*S*_	learning spatial scale	L	1
Movement^b^			
*τ*_*S*_, *τ*_*F*_	autocorrelation time scale	T	4, 2
*ν*_*S*_, *ν*_*F*_	length of *μ*	L/T	6, 1
*γ*_*Z*_	memory spatial scale	L	1, 5, 10
*λ*	mean time to update *θ*	T	1

^*a*^ *L* = long-term memory, *S* = short-term memory

^*b*^ *S* = searching, *F* = feeding

### Habitat quality and consumption

The habitat is modeled as a continuous scalar measure of resource quality that varies across the landscapes. The intrinsic quality, *Q*_0_(*z*), is constant through time, meaning that we do not consider transient or moving patches. The instantaneous habitat quality, *Q*(*z*, *t*) depends on both consumption, *C*(*z*, *t*), by the animal and a logistic regeneration, *R*(*z*, *t*),

$$\begin{array}{c} \frac{\partial Q}{\partial t}=\left(R-C\right)Q. \end{array}$$ (1)

Consumption and regeneration are defined as

$$\begin{array}{c} C=\beta_{C}\;f_{C}(|z-Z|), \end{array}$$ (2)

$$\begin{array}{c} R=\beta_{R}(1-\frac{Q}{Q_{0}}), \end{array}$$ (3)

where *β*_*R*_ is the regeneration rate, and consumption occurs at animal’s position, *z* = *Z*, described by an isotropic spatial kernel, *f*_*C*_(|*z* − *Z*|), and consumption rate, *β*_*C*_. We use a bivariate normal distribution for *f*_*C*_, and the variance parameter $\gamma_{C}^{2}$ (length scale) characterizes how widely the animal consumes about its location (Table 2). This conception of resource consumption, high in the animal’s immediate vicinity and decaying to zero at greater distances, is a good fit for grazing animals, with the length scale representing how far an animal can reach for food as it moves or fine-scale movements on a smaller scale than the trajectory [29]. Within our framework, however, any kind of kernel, including those with limited spatial extents such as discrete top-hat kernels, can be implemented as straightforwardly as others.

**Table 2:** Spatial kernels used in the foraging model. 𝒩_2_ is the bivariate normal distribution and ***I*** is the 2 × 2 identity matrix.

Description	Equation	Form
Consumption kernel	*f*_*C*_	${𝒩}_{2}(0,\gamma_{C}^{2}I)$
Short-term memory learning kernel	*f*_*S*_	${𝒩}_{2}(0,\gamma_{S}^{2}I)$
Long-term memory learning kernel	*f*_*L*_	${𝒩}_{2}(0,\gamma_{L}^{2}I)$
Memory distance-weighting kernel	*f*_*Z*_	Exp(*γ*_*Z*_)

### Memory map

As the animal moves across the landscape observing habitat quality, it builds up a memory map, *M*(*z*, *t*) made up of two streams. Two memory streams have been used to detect changes in the environment [30], combine short-term tactical and longer-term strategic behaviors in foraging [31], give rise to stable non-territorial home ranges [24], and represent distal and proximal expectations of reward in conditioning experiments [32]. Multiple memory layers have been suggested for modeling memory decay [11]. The memory map includes a long-term stream, *L*(*z*, *t*), which decays slowly and attracts the animal to high quality habitat, and a short-term stream, *S*(*z*, *t*), which decays more quickly and repels the animal from depleted habitat it has recently occupied. The two memory streams combine linearly to form the memory map, so that positive values are attractive, zero indicates neutrality, and negative values are repulsive:

$$\begin{array}{c} M=L-\psi_{M}S. \end{array}$$ (4)

Because *L* and *S* have the same maximum value (*Q*_0_) and *S* decays faster than *L*, the short-term memory factor, *ψ*_*M*_, ensures that the value at a just-visited location will initially be negative, or repulsive, with *ψ*_*M*_ > 1. As *L* and *S* decay, the value eventually turns positive and thus attractive for good quality habitat.

Each memory component (*L* and *S*) is a mixture of two parts, learning and forgetting,

$$\begin{array}{cc} \frac{\partial L}{\partial t} & =\beta_{L}f_{L}(|z-Z|)\left(Q_{0}-L\right)-\phi_{L}L, \end{array}$$ (5)

$$\begin{array}{cc} \frac{\partial S}{\partial t} & =\beta_{S}f_{S}(|z-Z|)\left(Q_{0}-S\right)-\phi_{S}S, \end{array}$$ (6)

where *β*_*L*_ and *β*_*S*_ are the learning rates of the long- and short-term memory streams, *f*_*L*_ and *f*_*S*_ are spatial kernels describing learning (Table 2), and *ϕ*_*L*_ and *ϕ*_*S*_ are the decay rates (*ϕ*_*L*_ < *ϕ*_*S*_).

### Movement model

#### Movement process

An animal’s movements through a landscape are described by a continuous trajectory, *Z*(*t*). Taking velocity, *V*(*t*), the animal’s position is thus $Z(t)=\int_{0}^{t}V(t^{\prime })dt^{\prime }+Z(0)$, where *Z*(0) is the animal’s initial position. An autocorrelated, directed, continuous movement process is

$$\begin{array}{c} dV=\frac{1}{\tau }\left(\mu \left(t\right)-V\right)dt. \end{array}$$ (7)

This is similar to the Ornstein-Uhlenbeck process, but without the white noise component. Instead, stochasticity is introduced through the bias vector, described by its magnitude and angle as *μ*(*t*) = (*ν*, ∠*θ*). The movement process is parameterized by *τ*, the time scale of autocorrelation, and *ν* = ||*μ*(*t*)||, the magnitude of the bias vector which controls the average speed of the process. The angle *θ* is set probabilistically, either from a uniform circular distribution resulting in a random walk or from a probability distribution computed from the memory map. A Poisson process with rate parameter *λ* is used to update *θ*, and *μ*(*t*) is constant between updates.

#### Behavior states

Three nested versions of the movement process are compared: random walk, kinesis, and memory. For the single-state random walk model, a single set of speed and time scale parameters determine the movement process. With the kinesis and memory models, the animal switches between searching and feeding states (Table 3). These three movement processes have the advantage of being nested, facilitating comparisons. Thus, random movement is a special case of kinesis where both behaviors have identical parameter values, and kinesis in a special case of memory with either zero learning rates or immediate decay rates. Kinesis is a movement model that performs well in a variety of environments and avoids the strong assumptions of perceptual abilities of area-restricted search [19], which is also known to perform sub-optimally in very patchy environments [33]. Random walk provides a useful null model to compare against.

**Table 3:** Behavioral states in the model and corresponding movement process parameters.

State	Direction (∠*μ*)	Speed (||*μ*||)	Time scale (*τ*)
Searching	memory: ∠*μ* ∼ *g*(*θ*) kinesis: ∠*μ* ∼ *U*(0, 2*π*)	*ν*_*S*_ (fast)	*τ*_*S*_ (large)
Feeding	∠*μ* ∼ *U*(0, 2*π*)	*ν*_*F*_ (slow)	*τ*_*F*_ (small)

For both kinesis and memory models, movement in the feeding state is tortuous and slow as the animal seeks to exploit the local high quality habitat. Movement is undirected, so the angle *θ* of the bias term is drawn from a uniform distribution with update times determined by the Poisson process. The searching state, on the other hand, is characterized by more linear movements and directional persistence, and the animal moves with a faster speed. Bias angles still change randomly for kinesis, but with memory, the animal seeks productive patches with the direction determined from the memory map. The angular probability distribution is computed by integrating transects of the memory map radiating out from the forager’s location with the memory value at each point weighted by distance. The integrated transects are then normalized by the integrated value for the whole memory. The angular probability density function is given by

$$\begin{array}{c} g\left(\theta \right)=\frac{\int_{0}^{r}M\left(r,\theta \right)f_{Z}\left(r\right)dr}{\int_{0}^{2\pi }\int_{0}^{r}M\left(r,\theta^{\prime }\right)f_{Z}\left(r\right)drd\theta^{\prime }}, \end{array}$$ (8)

where *r* = |*z* − *Z*| and *f*_*Z*_(*r*) is a kernel function (e.g., exponential with length scale parameter *γ*_*Z*_) that weights according to distance, such that closer resources are preferred all else being equal (Table 2).

#### Behavioral state transitions

The animal begins in the searching state, transitions to the feeding state when consumption increases, and transitions back to searching whenever consumption drops. Following the marginal value theorem of optimal foraging theory, an animal should leave a patch when the foraging rate in the patch drops below the average foraging rate in the environment [34]. Thus, in our simulations, the switch between states occurs when the instantaneous consumption rate, *C*(*t*), crosses the average consumption rate, $\bar{C}$. The average consumption rate is based on the intrinsic habitat quality, $\bar{C}=\beta_{C}\;f_{C}(|z-Z|)\bar{Q_{0}}$, where $\bar{Q_{0}}$ is the average habitat quality over all space.

### Simulations

We ran tens of thousands of simulations of the foraging process based on all three movement models across a range of landscape properties according to the parameter values in Table 1. The parameter space was explored systematically, with simulations run for all combinations of specified parameter values. For each movement model, we ran twenty replicate simulations for each combination of the landscape parameters of patch concentration, patch size, and regeneration rate. For the memory model, each combination of memory parameters were run across all landscape replicates.

To make the simulations as comparable as possible, we used the same speed and timescale autocorrelation parameters for both the memory and kinesis models, with the random walk model using the faster, more linear searching behavior speed and timescale autocorrelation, i.e., the random movement parameterization which leads to the highest foraging efficiency as per encounter theory [35].

To examine a stationary scenario (i.e., after the animal is familiar with its environment), simulations begin with the long-term attractive memory stream initialized to the habitat quality, *Q*_0_, and the short-term repulsive stream initialized to zero. Similarly, for the kinesis and memory models, the animal is assumed to know the average consumption rate, $\bar{C}$. This assumption corresponds to a forager that is broadly familiar with its environment of regenerating resources, in contrast to a completely naive (e.g., introduced) forager.

The continuous time model (Eqs 4–8) was implemented in Java, with time discretized with small regular intervals Δ*t* approximating *dt*. If an event from the Poisson process that updates *μ*(*t*) occurred during the interval, then a new angle *θ* was selected at that interval. The differential equations described above were approximated using the Euler forward method [36]. The time step was selected to satisfy the requirements of the Courant–Friedrichs–Lewy criterion [37] that the time step be smaller than the spatial resolution divided by the magnitude of the fastest velocity to ensure the accuracy and stability of the approximation and also to satisfy Δ*t* < *τ* (Table 1).

#### Landscapes

**Fig 1:**
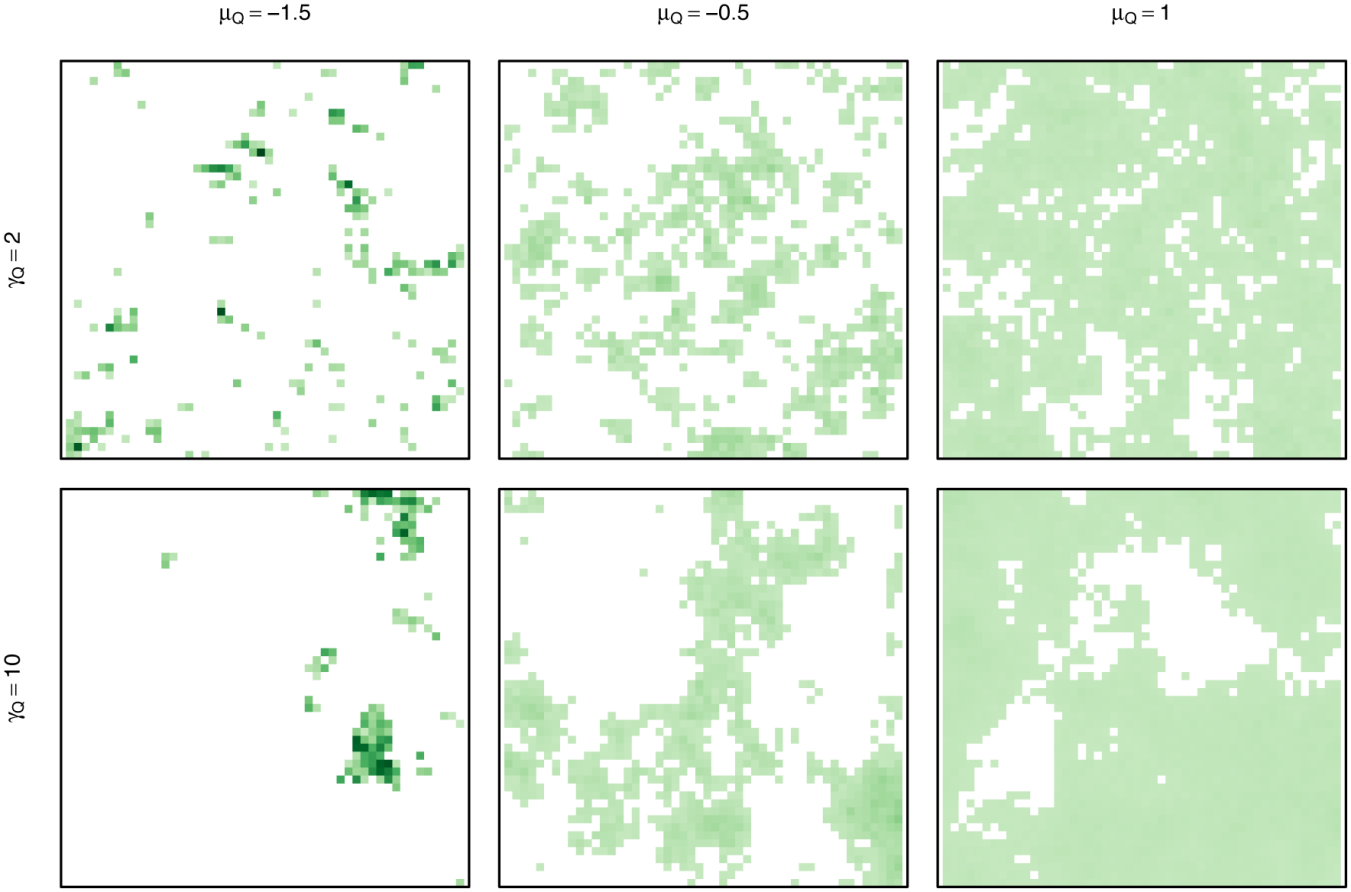
Sample generated landscapes for different combinations of patch concentration from patchy to smooth and patch size from small to large. Color indicates resource quality from none (white) to low (light green) to high (dark green). Total resources in each landscape are the same.

To simulate the model, the continuous variation in habitat quality is discretized onto a grid. Landscapes were generated with a Gaussian random field (GRF) using the RandomFields R package [38] to be 50 × 50 in size using an exponential covariance function with variance = 1, nugget = 0, and a set of mean (patch concentration, *μ*_*Q*_) and scale (patch size, *γ*_*Q*_) values (Fig 1, Table 1). Differing levels of patchiness were simulated by truncating all negative values to zero, resulting in more areas of no resources for smaller mean values. Landscapes were normalized to sum to one so that the total resources are the same across all landscapes. For each combination of *μ*_*Q*_ and *γ*_*Q*_, twenty landscape replicates were generated.

The GRF mean parameter, or *patch concentration*, controls how concentrated resources are in space along a gradient from patchy (sparsely located high-value resources) to smooth (widespread lower-value resources). The GRF scale parameter, or *patch size*, controls the relative size and continuity of patches in the generated landscape via the spatial autocorrelation in the generated field. Note that the landscape parameters are not completely orthogonal: patchier landscapes have smaller patches than smooth landscapes. These parameters allowed us to explore a range of landscape types, though the behavioral model is not tied to these generated landscapes.

The initial location of foragers was in all cases the midpoint of each landscape, and the boundaries were reflective. In the memory case, the *μ* vector was reset on hitting a boundary, either randomly for kinesis or according to the angular probability density function for memory, but restricted to the quadrants away from the boundary. While the memory model can lead to home range behavior [24], the kinesis and random walk models are dispersive, and thus using a bounded landscape makes these processes more comparable while providing a conservative estimate for any advantage from memory.

#### Metrics

The primary metric used to evaluate simulations was total consumption, i.e., the sum of the forager’s consumption (Eq 2) over the fixed duration of each simulation (1000 time steps). Additional metrics were habitat usage (time spent in areas of zero resources and the four quartiles of resources for areas with positive quality) and time spent in each behavioral state, searching and feeding, for kinesis and memory movement processes.

The analysis of model outputs was done with R [39]. To compare the three movement processes we performed the Approximative K-Sample Permutation test [40] where post-hoc comparisons were made with the Nemenyi-Damico-Wolfe-Dunn (NDWD) test using the coin R package [41, 42] and a p-value <0.05 considered significant. Comparisons were made in aggregate, pooling both memory and landscape parameterizations. We also compared the movement processes for each memory model parameterization across all the landscape paramterizations. In these cases we adjusted the p-values to control the false discovery rate using the Benjamini–Hochberg (BH) procedure [43]. To evaluate the contribution of different parameters in the memory model, we used random forests to compute a statistic of relative importance for each parameter [44] using the party R package [45]. An advantage of random forests is their robustness to nonlinearity and complex interaction effects [45]. The method first permutes one of the predictors (thereby removing the potential association between that predictor and the response), then generates random forests (a set of classification trees fit to bootstrap samples drawn from the original), and compares the prediction accuracy of the permuted and unpermuted predictor.

## Results

### Foraging efficiency

**Fig 2:**
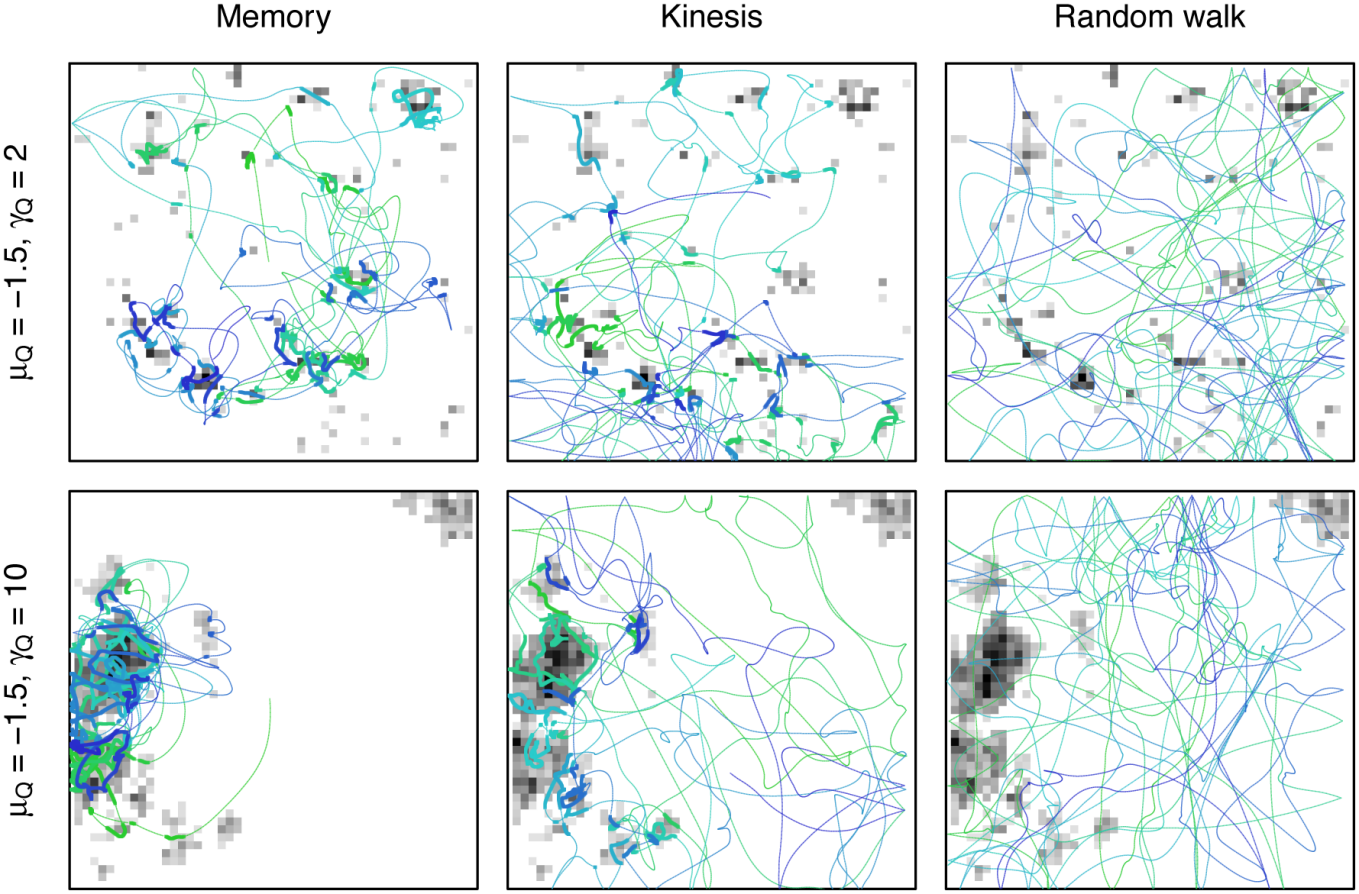
Sample trajectories for three movement models (left to right) on two different landscapes (top to bottom). Trajectories start at the center with color changing through time (from green to light blue to dark blue). For memory and kinesis, thin lines indicate searching and thick lines indicate feeding behavior. Resources are shown at their undepleted level at the beginning of the simulation. Memory is parameterized with best overall parameters, *ϕ*_*L*_ = 1*e* − 05, *ϕ*_*S*_ = 0.01, *ψ*_*M*_ = 2, *γ*_*Z*_ = 10.

Differences in simulation trajectories and space use, which translate to differences in consumption, were apparent (Fig 2). Averaging over all memory parameterizations and landscapes, the memory model (consumption mean = 0.43, s.e. = 0.29, n = 90720) outperformed the kinesis model (consumption mean = 0.27, s.e. = 0.11, n = 360) and the random walk model (consumption mean = 0.18, s.e. = 0.03, n = 360) and was significant across all groups (Approximative K-Sample Permutation test: maxT = 30.67, p <0.0001; NDWD post-hoc tests: p <0.0001 for all group comparisons). When examining each memory model parameterization separately against the kinesis and random walk models for each landscape parameterization, the total consumption for the three models was nearly always significantly different (98.7% Approximative K-Sample Permutation tests significant with BH p-value adjustment; S1 Appendix). Every memory model parameterization had higher mean consumption than the random walk model across all landscape parameterizations, and 98.7% of these were significantly different (NDWD post-hoc tests with BH p-value adjustment; S1 Appendix). Of the 67.2% of memory model parameterizations that were significantly different from the kinesis model (NDWD post-hoc tests with BH p-value adjustment; S1 Appendix), the memory model had higher average consumption in all cases. Thus, while there was more variability across simulations for the memory model, total consumption was also higher, meaning the increased variability with memory was towards improved performance.

**Fig 3:**
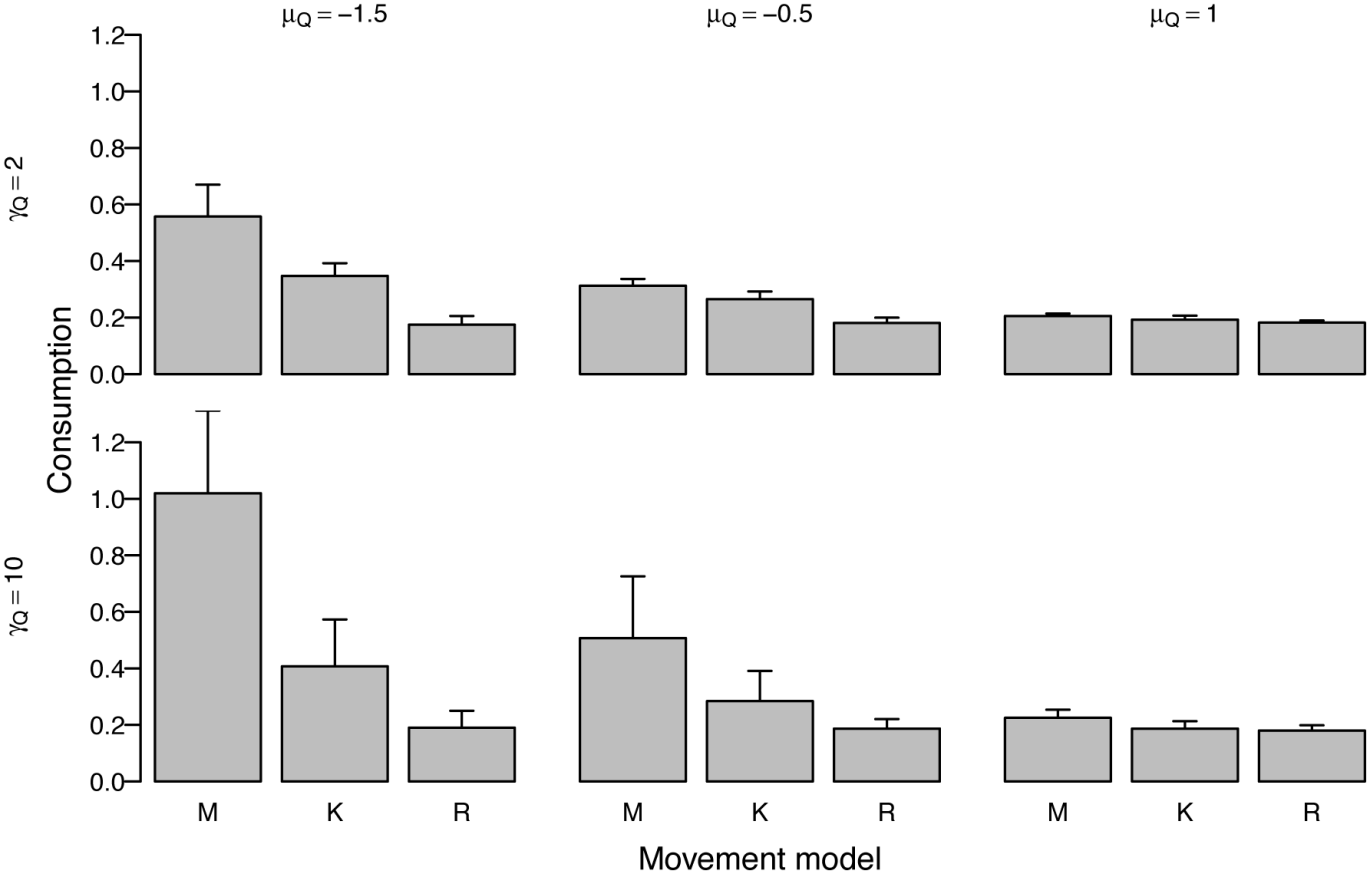
Consumption for the three movement models across different landscape parameters patch concentration *μ*_*Q*_ and size *γ*_*Q*_ for medium regeneration rate *β*_*R*_ = 0.01. Bars show mean consumption values across replicates of landscape parameters while lines show minimum and maximum. Memory is parameterized with best overall parameters, *ϕ*_*L*_ = 1*e* − 05, *ϕ*_*S*_ = 0.01, *ψ*_*M*_ = 2, *γ*_*Z*_ = 10. In the figure, M = memory, K = kinesis, R = random walk.

Landscape characteristics affected the degree of benefit memory provides (Fig 3). In more patchy environments (negative *μ*_*Q*_) with larger patches (large *γ*_*Q*_), the memory model (using the best overall parameterization across all landscapes) strongly outperformed the kinesis model, which in turn outperformed the random walk model, although the effect weakened as the landscapes become smoother (positive *μ*_*Q*_) and patches smaller (small *γ*_*Q*_). Similarly, the percent of memory parameterizations for which consumption was significantly different from the kinesis model was highest in the best performing landscapes (S1 Fig). The pattern held across regeneration rates, but was strongest for higher regeneration rates. Mean consumption values remained constant across landscape parameterizations for the random walk model, increased with only increasing patch concentration for the kinesis model, and increased with both increasing patch concentration and size (*γ*_*Q*_) for memory. Variability across simulations also increased for all movement processes with increasing patch concentration and size (i.e., landscapes with fewer larger patches). Even with increased variability in landscapes with high-value and/or large patches, the minimum memory model consumption was higher than the maximum random walk model and generally higher than or close to the mean kinesis model value.

### Memory parameters

To assess the best performing memory parameter combination for each landscape parameter combination, we compared the total consumption averaged over landscape replicates (Table 4). While there was less variability within memory parameterizations than across movement processes, patterns still emerged. The best performing short decay rate varied consistently with regeneration rate. There was less consistency in the long decay rate. Interestingly, a long decay rate of 0 was rarely selected, though resource stability means there is no obvious advantage to decaying the long-term attractive memory stream. Little pattern was apparent with the short memory factor in relation to the landscape parameters, although there appeared to be an interaction between the short decay rate and the short memory factor. Larger short memory factors were associated with relatively faster short decay rates. Thus the short-term repulsive memory stream may similarly adjust the overall memory by either a lower weighting of a slowly decaying memory or a higher weighting of a faster decaying memory (Eqs 4 and 6). Finally, a large value was favored for the memory spatial scale, i.e., giving more weight to distant patches.

**Table 4:** Best performing memory parameters (*ϕ*_*L*_, *ϕ*_*S*_, *ψ*_*M*_, *γ*_*Z*_) for each landscape environment, a combination of regeneration rate (*β*_*R*_), patch concentration (*μ*_*Q*_), and patch size (*γ*_*Q*_).

		*β*_*R*_ = 0.005				*β*_*R*_ = 0.01				*β*_*R*_ = 0.05			
*μ*_*Q*_	*γ*_*Q*_	*ϕ*_*L*_	*ϕ*_*S*_	*ψ*_*M*_	*γ*_*Z*_	*ϕ*_*L*_	*ϕ*_*S*_	*ψ*_*M*_	*γ*_*Z*_	*ϕ*_*L*_	*ϕ*_*S*_	*ψ*_*M*_	*γ*_*Z*_
-1.5	2	1e-05	0.01	2	10	1e-04	0.1	5	10	0.01	0.5	10	5
-1.5	10	1e-05	0.01	2	10	0.001	0.5	5	10	1e-04	0.5	2	10
-0.5	2	1e-04	0.01	10	5	1e-04	0.1	10	5	0.01	0.5	5	5
-0.5	10	1e-05	0.01	2	10	0	0.1	5	5	1e-05	0.1	5	10
1	2	0.001	0.1	10	5	0.01	0.1	10	10	0.01	0.1	5	10
1	10	0.01	0.1	5	10	0.001	0.1	2	10	0.001	0.1	10	10

Comparing variable importance to explain the differences in consumption, landscape parameters dominated memory parameters (Table 5). A conservative rule of thumb to interpret variable importance values is that a variable is informative if its value is greater than the absolute value of the lowest negative value, as irrelevant predictors will randomly vary around zero [46]. All parameters had positive variable importance values. Of the landscape parameters, patch concentration was the most important, followed by patch size and regeneration rate at a similar order of magnitude. For the memory parameters, the short decay rate was most important, followed by memory spatial scale at a similar order of magnitude, then short memory factor and long decay rate. The long decay rate may only be important as a threshold (i.e., slow enough), and the bulk of the observations were for smaller long decay rates.

**Table 5:** Permutation importance scores (mean decrease in accuracy) calculated using random forests for the memory model. Results shown treat parameters as continuous variables. Results were similar when parameters were treated as categorical.

Parameter		Variable importance (e-3)
*μ*_*Q*_	landscape patch concentration	96.3
*γ*_*Q*_	landscape patch size	27.4
*β*_*R*_	regeneration rate	6.32
*ϕ*_*S*_	short decay rate	2.65
*γ*_*Z*_	memory spatial scale	2.47
*ψ*_*M*_	short memory factor	0.310
*ϕ*_*L*_	long decay rate	0.0267

As another approach to evaluating memory, we examined different parameterizations for how often they were significantly different from the kinesis model (S2 Fig) and how much improvement over kinesis (S3 Fig) they provided (memory parameterization nearly always outperformed the random walk model). The clearest pattern emerged with memory spatial scale, *γ*_*Z*_, with larger values outperforming smaller values almost exclusively, where larger values mean less discounting of distant resources in memory. For the short decay rate, *ϕ*_*S*_, larger values, and thus faster decay, were generally better, while the opposite was true for the long decay rate, *ϕ*_*S*_. There was some clustering evident with the short memory factor, *ψ*_*M*_, as well. For specific landscape parameterizations, the general patterns in parameter values still held but the magnitude of difference between the kinesis and memory models mirrored Fig 3.

### Behavior

**Fig 4:**
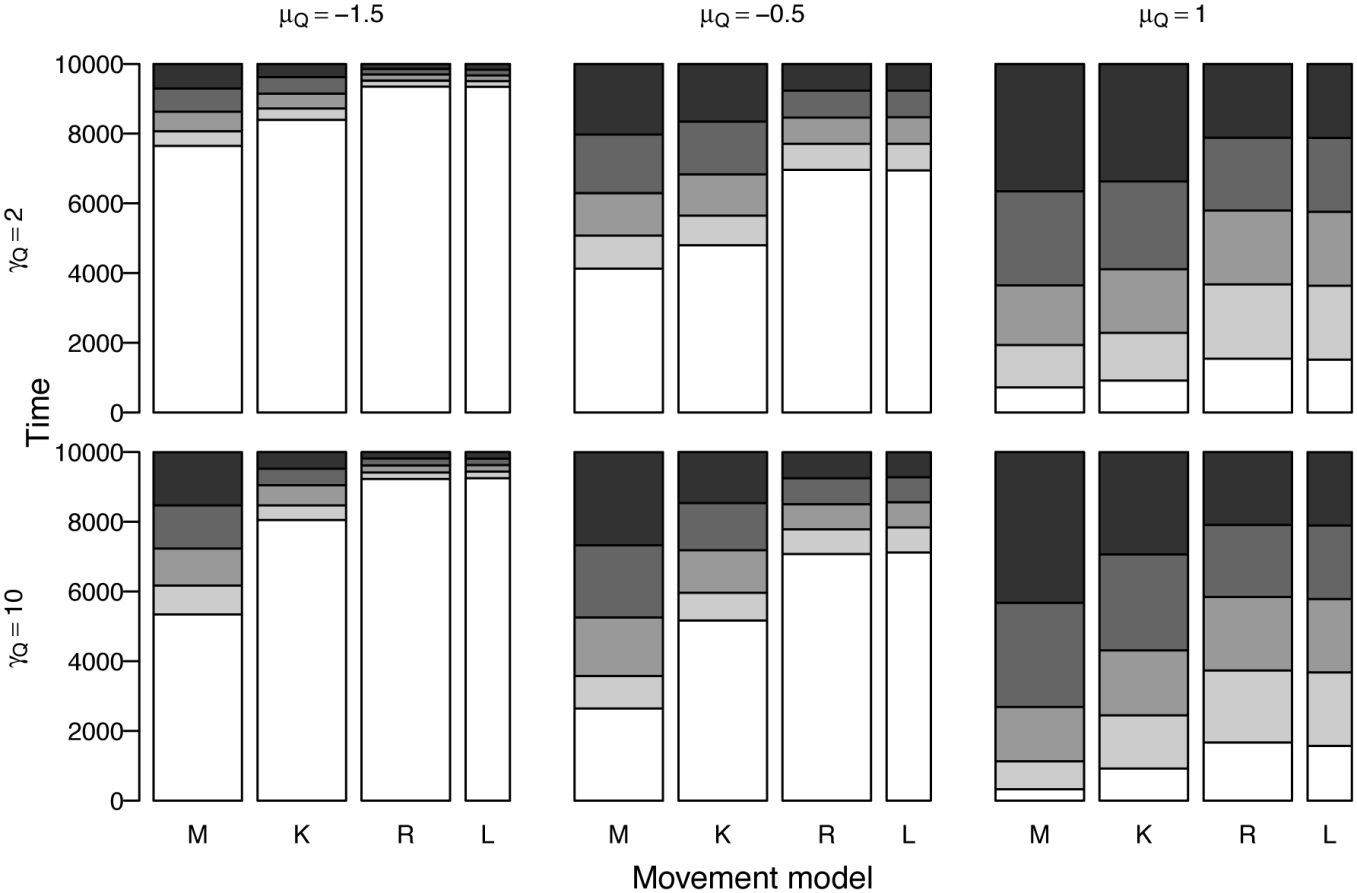
Time spent in areas of different resource quality across different landscape parameters patch concentration *μ*_*Q*_ and size *γ*_*Q*_ for medium regeneration rate *β*_*R*_ = 0.01 compared to the distribution of resources on the landscape. White represents zero resources while shades of gray from light to dark show quartiles of increasing quality. The memory model is parameterized with best overall parameters, *ϕ*_*L*_ = 1*e* − 05, *ϕ*_*S*_ = 0.01, *ψ*_*M*_ = 2, *γ*_*Z*_ = 10. In the figure, M = memory, K = kinesis, R = random walk, L = landscape.

The differences in habitat usage observed across the movement processes for different landscape parameterizations (Fig 4) mirrored the differences in spatial concentration for the individual trajectories (Fig 2). The amount of habitat with zero resources varied with patch concentration, from most areas having zero resources (*μ*_*Q*_ = −1.5) to some positive amount of resources nearly everywhere (*μ*_*Q*_ = 1). As expected, the habitat usage of the random walk model matched the distribution of habitat on the landscape. For the kinesis model, habitat usage was markedly biased towards areas of higher quality, though this was not affected by patch size, *γ*_*Q*_. For the memory model, on the other hand, habitat usage was more skewed towards better quality areas than the kinesis model and also differed by patch size as well as concentration.

**Fig 5:**
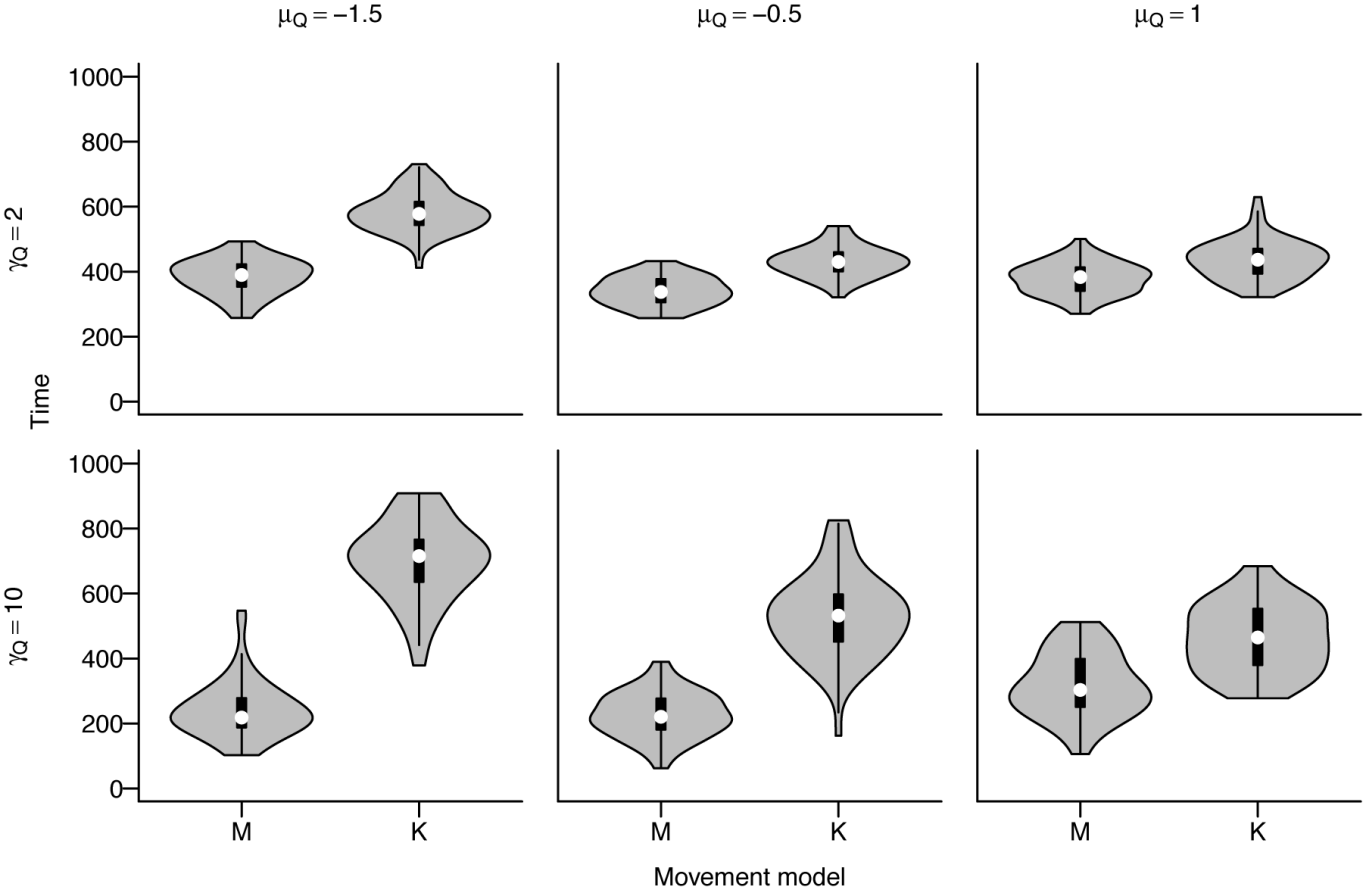
Time spent searching (as opposed to feeding) for memory and kinesis models across different landscape parameter values for patch concentration *μ*_*Q*_ and size *γ*_*Q*_ for medium regeneration rate *β*_*R*_ = 0.01 as a violin plot showing median values and kernel density plot. The memory model is parameterized with best overall parameters, *ϕ*_*L*_ = 1*e* − 05, *ϕ*_*S*_ = 0.01, *ψ*_*M*_ = 2, *γ*_*Z*_ = 10. In the figure, M = memory, K = kinesis.

The memory-informed foragers spent less time searching on average than the kinesis-driven foragers (Fig 5). In the kinesis model, time searching decreased with smoother landscapes where most areas contained some resources. Search time for the memory model was generally consistent across differing patch concentration values, increasing only slightly in patchier landscapes. On the other hand, the kinesis model showed increasing search time variability with increasing patch size, but the median time searching was similar. For the memory model, time searching decreased substantially with increasing patch size.

## Discussion

### Comparative analysis of model implementation and outcomes

We developed a continuous-time, continuous-space foraging model that incorporates movement with directional preference based on a memory of habitat quality that allowed us to examine the effect of landscape characteristics on the performance of different movement processes, particularly memory. Our model simulation exercises suggest that while the different movement processes perform similarly in smooth landscapes, more complex processes perform better in patchier landscapes. The concentrated resources in patchier landscapes are both harder to locate and of higher value once located, leading to the memory model outperforming the random walk model and the kinesis model. Similarly, foragers using memory also receive higher rewards with a faster regeneration rate and larger high-value patches, which also provide a stronger signal in memory. In general, landscapes that favor memory are those with resources that are higher value or more difficult to encounter, and these landscapes are the places best-suited to look for evidence of memory-informed foraging behavior.

The three movement processes modeled here are nested, so the same underlying movement model is used. Additional behavioral complexity, like switching states or using memory-informed directional biases, can be enabled or disabled within the same framework. This allows for better comparisons between movement processes for simulation studies and also for hypothesis testing if the model is fit to data. The memory implementation is both simple and conforms to current knowledge in animal cognition. Two memory streams are a common feature of cognitively-based ecological models [24, 30–32] and reflect evidence for multiple parallel memory systems that interact [47, 48].

We utilize a continuous space and time architecture in the the model to compare processes that occur across scales, e.g., a single lever can transition between highly tortuous and highly linear movement, between highly patchy and highly homogenous landscapes, and between highly local and highly distributed spatial kernels of foraging and memory. For the movement process particularly, a continuous framework mimics the actual continuous movement of an animal across a continuous landscape, even though sampled discretely. Recent work has focused on parameterizing continuous movement processes from trajectories [49–51] and using continuous-space formulations in spatial population models to represent spatial heterogeneity from multiple sources and at multiple scales [1]. While discrete correlated random walks (CRW) are a common modeling approach (e.g., [14, 21, 24]), CRWs are highly scale-dependent with difficulties in both reformulating from one interval to another and selecting the interval [52, 53]. Discrete movements between patches is one clearly observable and common behavior. This process is modeled, approximately, by the behavioral changes inherent in the behavioral switches of the memory model. Indeed, a discrete movement between known patches under certain conditions is an emergent property of the memory model. However, the model as currently formulated does not accommodate many features of animal movement, such as diurnal behavior. Additionally, we acknowledge that there are problems with scaling at the extremes with both very large and infinitesimal scales.

Using spatially continuous landscape generated from Gaussian random fields allows us to explore a continua of resource patchiness and heterogeneity. However, the behavioral model is completely independent of the landscape, and it can be run on a real landscape based on sampled or remotely sensed data (e.g., [20, 54]), or a landscape of discrete patches (e.g., [24, 26, 35]). An advantage to a continuous landscape representation is that field data are virtually always continuous in some way, while patch-based approaches often make unrealistic assumptions about the shape of the patches and are thus harder to apply to field data. However, this representation is not necessarily applicable to all situations, such as fruiting trees [21] or mobile prey [55]. However, other representations for the landscape and foraging process could be used with the continuous movement model, including discrete models more appropriate to some systems that can almost always be generated as special cases of the continuous models we present. Additionally, the model simplifies the environment into a single gradient, while real animals nearly always have to balance tradeoffs between different requirements and therefore do not move on a single gradient landscape. Moreover, most ecological applications do not directly measure the distribution of an animal’s resources on the landscape but instead use remotely-sensed proxies for those resources (e.g., the normalized difference vegetation index (NDVI) as a proxy for forage quality [56]).

Our use of continuous Gaussian kernels in the foraging and memory processes are perhaps more limited in their applicability. The local distance-dependent kernel for learning is analogous to the detected local environment (e.g., via vision or smell), while the kernel of foraging can be thought of as local foraging movements that occur on a finer scale than the displacements, though these analogies break down at infinitesimal scales. Nonetheless, in either case, any kind of kernel can be implemented as straightforwardly as others within our framework, including smaller, non-infinite, or discrete (top-hat) kernels. Other limitations of the model include the exclusion of processes such as bioenergetics, including starvation due to the temporal pattern of energy consumption, stochasticity in resource acquisition or handling time, and diurnal or resting behavior. While all these would be possible to incorporate in the model, we chose to omit them for simplicity. Instead, we focused on the movement, foraging, and memory processes necessary to address our main question of how landscape characteristics affect the performance of the memory model compared to other movement processes.

Our findings corroborate prior work, even where the conception of memory is different. Grove’s [27] conclusion that memory is more useful for lower patch density is analogous to the result here for patchier landscapes (with less area containing resources). Our finding that larger patches decrease the utility of memory is less directly comparable due to differences in landscape construction, but the qualitative conclusion that memory is less useful when patches are more easily discoverable is supported. In models that investigate learning and memory of consumption and encounter rates, rather than the spatial location of resources, patch heterogeneity is also an important predictor of the forager’s consumption, with memory most useful in conditions of resource heterogeneity and a long-term memory allowing the forager to concentrate effort in the highest-value patches [57–59]. Avgar et al. [15] suggest only physiological constraints should limit memory in an unchanging environment, but that forgetting is adaptive in a temporally changing environment, with memory capacity negatively correlated to the rate of change. Here, the available amount of resources varied temporally, and the rate of forgetting (the short-term memory decay rate) tracked the rate of environmental change (the regeneration rate). The utility of memory in high complexity landscapes may be reduced due to the high cost of tracking sufficient information [11]. An interesting extension of this work would be to model memory at different spatial resolutions and determine if an increasing cost for more detailed memory would produce the dome-shaped relationship between memory utility and resource complexity predicted by Fagan et al. [11].

### Landscape characteristics

Landscape characteristics (Fig 1) mediate the magnitude of consumption variation among the three movement processes. In smooth, continuous landscapes with a relatively even distribution of low resources, the movement processes perform similarly. In patchier landscapes with more area devoid of resources, the kinesis model generally outperforms the random walk model and the memory model outperforms the kinesis model. Resources are both more difficult to locate and of higher value in patchier landscapes, as we held the total amount of resources constant across landscapes. Patchy landscapes favor memory as the benefit of finding resources is high and memory directs movement towards hard-to-find patches. Any tendency to slow down in the few higher-valued patches is an advantage, however, as seen by the kinesis model outperforming the random walk model. The kinesis model has perfect knowledge of the average consumption rate, while if the consumption rate must be learned, the ability to exploit high-value patches increases with memory length [58].

Patch size is also important to consumption rates, as it informs the likelihood that nearby locations also have resources. As patch size increases, memory-based movement dramatically outperforms the kinesis and random walk models, though larger patches can also lead to higher consumption in perceptually-guided foragers [60]. Differences in search times highlight these differences: the memory model uniquely spends less time searching with increasing patch size and exploits the larger, more contiguous patches more efficiently, spending less time traveling between them. Travel time between patches is expected to differ between perceptually-guided and omniscient foragers [27], and this is an emergent property of our model. Larger patches give a stronger signal to the probabilistic direction (Eq 8). The memory-guided forager can easily return to a large patch after wandering off, but the forager using kinesis depends on randomly encountering patches again. Only consumption variability increases with patch size for the kinesis and random walk models, while both consumption and consumption variability increase with patch size for memory. Models struggle to capture the variability present in natural systems, and it has been suggested that spatially explicit models are necessary for foraging behavior to account for the effects of heterogeneous resource distribution and to allow for non-random walk strategies [59]. Although memory-informed movement can give rise to more predictable movement paths such as home range behavior [24, 25] and repeated routes [21], we suggest that it can also lead to increased variability in consumption and thus forager condition.

Performance of memory-based movement also depends on the interaction between the spatial scale of memory and patch sizes in the landscape. Larger memory spatial scales allow the forager to make movements towards distant locations [18]. The better performance of large memory spatial scales show the benefit of considering food farther away. Exceptions occur with small patch sizes, where small spatial ranges may perform better (Table 4). This may be due to small patches having smaller signals in memory, making it advantageous to weigh close resources higher. Small, distant patches may not be worth the travel time. Including perception [14] would likely amplify this effect, as more discontinuous patches would be harder to detect and exploit. Some results suggest that intermediate perceptual abilities perform best with heterogeneous resource distribution as foragers must balance habitat exploration with tradeoffs between time spent searching versus feeding [61].

Lastly, landscape regeneration impacts the performance of the alternative movement processes, something that has been considered in some models [24, 25], but is frequently omitted even in models that explicitly include memory [14, 59]. Differences become readily apparent with high regeneration rates. When patches regenerate quickly there is a benefit to returning soon, favoring memory. This has been demonstrated in other models, with slow regeneration rates resulting in the forager exploring the entire environment and fast regeneration rates resulting in home range behavior [25, 26].

Among the landscape characteristics, regeneration has the largest effect on the memory model’s optimal parameterization (Table 4). Faster short-term memory decay is preferred as the regeneration rate increases, returning the animal sooner to productive patches. Animals may track an assessment of the lag time to return to a patch, either inherited or learned based on environmental conditions. However, memory generally outperforms the other movement processes at non-optimal decay rates in patchy landscapes, suggesting that the performance of the memory model could be robust to regeneration rates varying seasonally or among resource types without tracking an assessment of patch return intervals.

### Potential applications

Our model accounts for environmental change through the regeneration rate and depletion by foragers, suggesting its potential application to systems with temporally varying heterogeneous resources, though our reliance on consumption as a metric provides challenges to model calibration given the difficulty of collecting such ancillary data to animal movements. Scales could vary spatially from a nectarivore foraging on flowering plants to a fish feeding in seagrass beds to a cervid browsing forest clearings. Scales could vary temporally from a plant producing additional flowers or fruit from one day to the next to more seasonal regeneration. For example, dugongs (*Dugong dugon*) forage on seagrass meadows that are patchily located in coastal waters, and the meadows themselves are spatially heterogeneous in quality based on biomass density and species composition [62]. Dugongs have been observed to revisit grazing locations with the return time correlating to regeneration time [63]. As evidence that memory can shape spatial population processes, dugong grazing pressure appears to drive community structure by creating favorable conditions for preferred species, a process known as cultivation grazing [63, 64]. Another candidate application is the Mongolian gazelle (*Procapra gutturosa*), a wide-ranging species [65], whose habitat preferences correlate to satellite-derived measures of habitat quality [56]. Gazelle movements are highly nomadic through a temporally dynamic landscape, leaving open the question of how much movements are driven by memory, perception, or randomness.

While evaluated separately here, the differing landscapes could also be thought of as different habitats an animal encounters throughout its life history. For example, different resource distributions could represent the differences between feeding and breeding habitats that an animal migrates between or the seasonally varying differences between summer and winter habitats. In this case, the total amount of resources may also differ, but the general conclusions in terms of the relative performance of different movement processes would still hold. Thus, if the model were tested in dynamic landscape conditions, it may be that memory is most useful in times of resource constraint, such as hard-to-find winter forage, after a habitat perturbation, or during times of more limited mobility, such as the breeding season. For example, ungulate species on the Isle of Rhum, Scotland all foraged on high-quality, high-biomass areas in the summer during high resource availability, but showed resource partitioning during the winter [66]. The decimation of foraging habitat caused by a cyclone and floods led to changes in dugong distributions and led to emigration to other areas [67].

In conclusion, we have developed a flexible framework for considering memory with a continuous-space and continuous-time movement model that could be applied to a variety of systems once calibrated and validated against field data. Our results suggest that the best environments to look for evidence of memory-driven search are those with sparse, contiguous patches of high-value resources that regenerate quickly located in an otherwise devoid landscape. Thus, there is a large payoff for finding a resource patch, whether in size, value, or locational difficulty. While separating memory-driven foraging from sensory-driven alternatives is difficult [11], our findings of disproportionate space use of higher value areas, higher consumption rates, and consumption variability all point to memory influencing the movement of animals in a variety of simulated contexts.

## Supporting Information

S1 Fig Percent of memory parameterizations which were significantly different from other movement models across landscape parameterizations. Filled characters show the percent of memory parameterizations which were significantly different that the kinesis model, and open characters show the percent of memory parameterizations which were significantly different from the random walk model (NDWD post-hoc tests with BH p-value adjustment; S1 Appendix). The memory model had higher consumption in all cases when it was significantly different from other models.
(TIF)

S2 Fig For each memory parameter, the percent of memory parameterizations which were significantly different from other movement models across values of that parameter. Thick lines show the percent of memory parameterizations which were significantly different that the kinesis model, and thin lines show the percent of memory parameterizations which were significantly different from the random walk model (NDWD post-hoc tests with BH p-value adjustment; S1 Appendix). The memory model had higher consumption in all cases when it was significantly different from other models.
(TIF)

S3 Fig Percent of simulations in which the memory model outperformed the kinesis model and net amount of consumption gained using memory. Each point represents a parameterization of the memory model. Simulations are matched by landscape and regeneration rate. Percent outperformed shows the percent of simulations for which the memory model outperformed the kinesis model for that set of parameters. Net percent improvement shows how much consumption improves with the memory model over the kinesis model. It is calculated by subtracting the amount consumed under the kinesis model from that consumed under the specific parameterization of the memory model divided by the total consumed by the kinesis model across all simulations. Panels are each color coded by different memory parameters.
(TIF)

S1 Appendix Results from Approximative K-Sample Permutation test and post-hoc comparisons with the Nemenyi-Damico-Wolfe-Dunn (NDWD) test. (CSV)

S2 Appendix Results from simulation model runs, including parameter combinations and total consumption over the simulation. (ZIP)
