# Supplementary figures and images for: Memory Effects on Movement Behavior in Animal Foraging
Source: PLoS One. 2015 Aug 19;10(8):e0136057. doi: 10.1371/journal.pone.0136057 (PMC4542208; doi:10.1371/journal.pone.0136057)

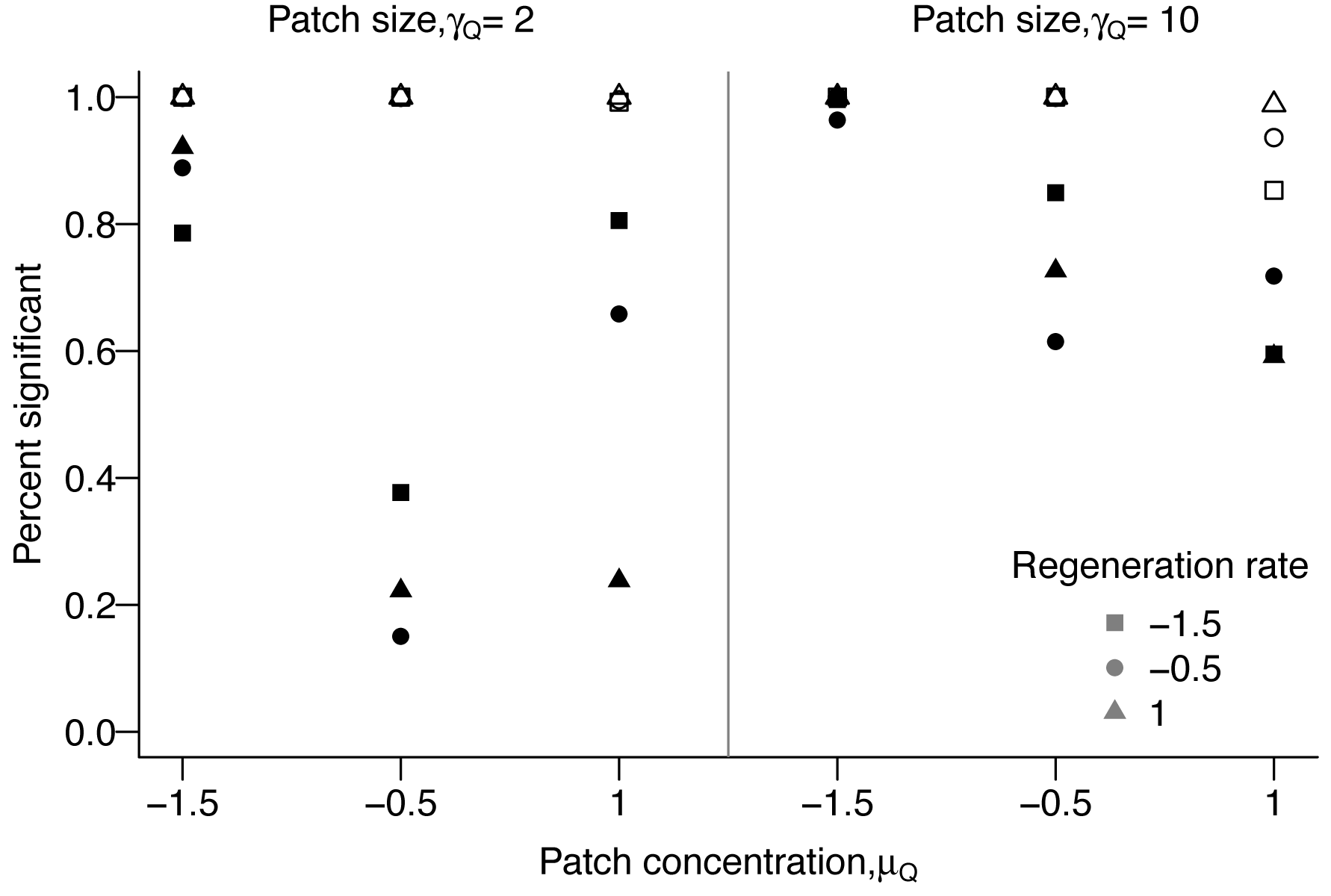

Supplement: S1 Fig — Filled characters show the percent of memory parameterizations which were significantly different that the kinesis model, and open characters show the percent of memory parameterizations which were significantly different from the random walk model (NDWD post-hoc tests with BH p-value adjustment; S1 Appendix). The memory model had higher consumption in all cases when it was significantly different from other models. (TIF) [file pone.0136057.s001.tif]

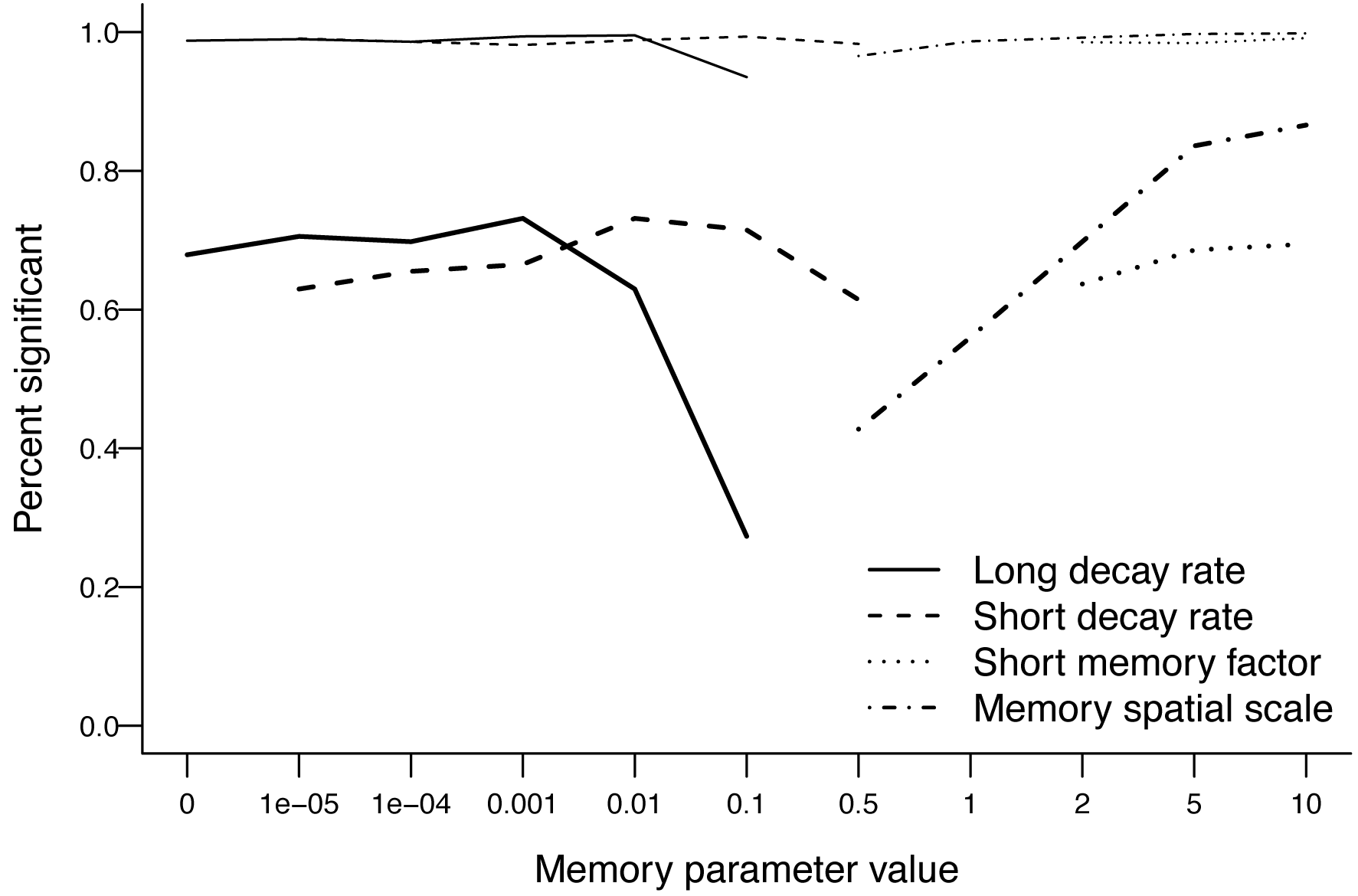

Supplement: S2 Fig — Thick lines show the percent of memory parameterizations which were significantly different that the kinesis model, and thin lines show the percent of memory parameterizations which were significantly different from the random walk model (NDWD post-hoc tests with BH p-value adjustment; S1 Appendix). The memory model had higher consumption in all cases when it was significantly different from other models. (TIF) [file pone.0136057.s002.tif]

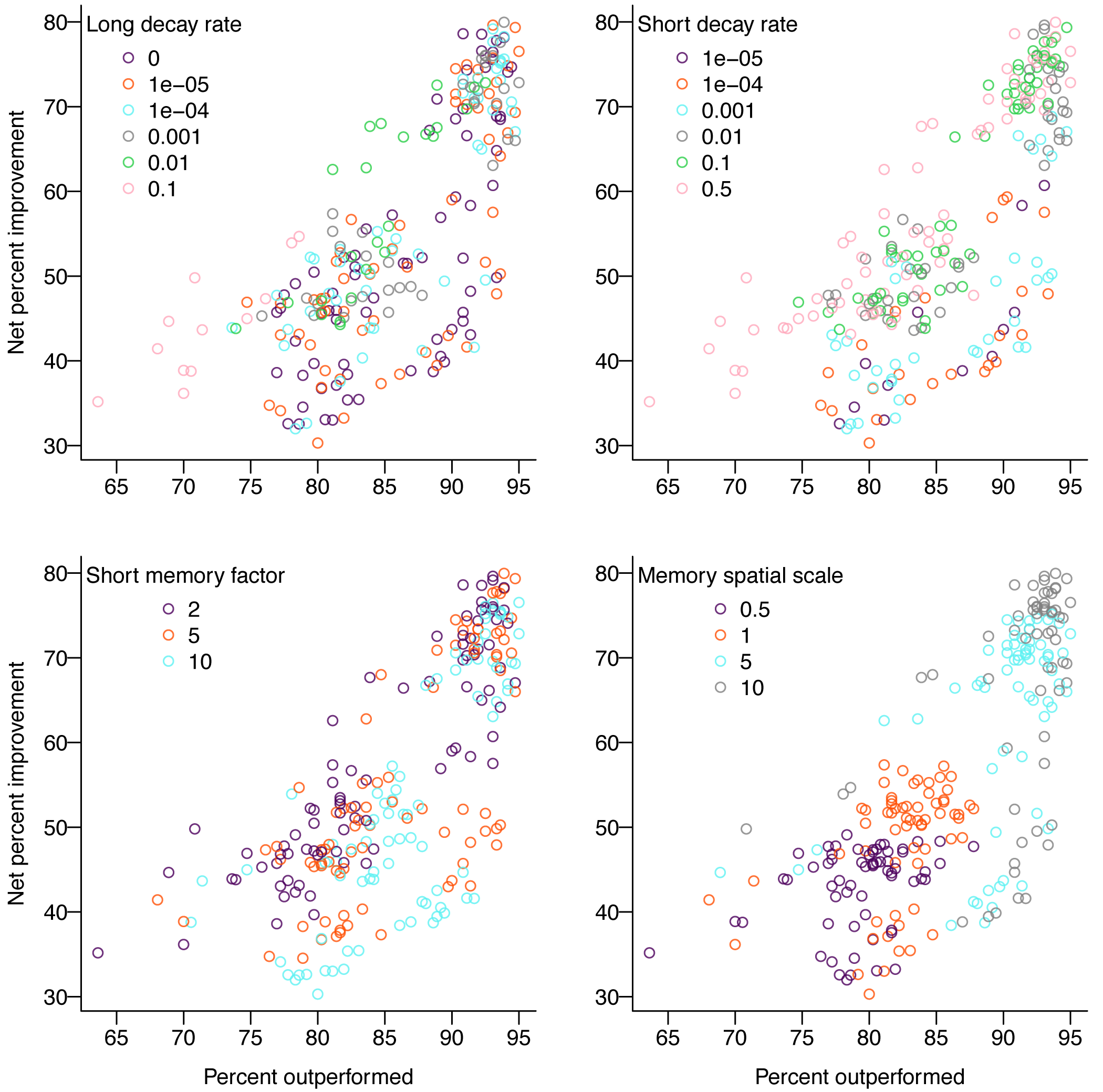

Supplement: S3 Fig — Each point represents a parameterization of the memory model. Simulations are matched by landscape and regeneration rate. Percent outperformed shows the percent of simulations for which the memory model outperformed the kinesis model for that set of parameters. Net percent improvement shows how much consumption improves with the memory model over the kinesis model. It is calculated by subtracting the amount consumed under the kinesis model from that consumed under the specific parameterization of the memory model divided by the total consumed by the kinesis model across all simulations. Panels are each color coded by different memory parameters. (TIF) [file pone.0136057.s003.tif]
